# Image-Based Medical Expert Teleconsultation in Acute Care of Injuries. A Systematic Review of Effects on Information Accuracy, Diagnostic Validity, Clinical Outcome, and User Satisfaction

**DOI:** 10.1371/journal.pone.0098539

**Published:** 2014-06-02

**Authors:** Marie Hasselberg, Netta Beer, Lisa Blom, Lee A. Wallis, Lucie Laflamme

**Affiliations:** 1 Department of Public Health Sciences, Karolinska Institutet, Stockholm, Sweden; 2 Division of Emergency Medicine, Faculty of Medicine and Health Sciences, Stellenbosch University, Cape Town, South Africa; 3 University of South Africa, Pretoria, South Africa; University of Groningen, University Medical Center Groningen, Netherlands

## Abstract

**Objective:**

To systematically review the literature on image-based telemedicine for medical expert consultation in acute care of injuries, considering system, user, and clinical aspects.

**Design:**

Systematic review of peer-reviewed journal articles.

**Data sources:**

Searches of five databases and in eligible articles, relevant reviews, and specialized peer-reviewed journals.

**Eligibility criteria:**

Studies were included that covered teleconsultation systems based on image capture and transfer with the objective of seeking medical expertise for the diagnostic and treatment of acute injury care and that presented the evaluation of one or several aspects of the system based on empirical data. Studies of systems not under routine practice or including real-time interactive video conferencing were excluded.

**Method:**

The procedures used in this review followed the PRISMA Statement. Predefined criteria were used for the assessment of the risk of bias. The DeLone and McLean Information System Success Model was used as a framework to synthesise the results according to system quality, user satisfaction, information quality and net benefits. All data extractions were done by at least two reviewers independently.

**Results:**

Out of 331 articles, 24 were found eligible. Diagnostic validity and management outcomes were often studied; fewer studies focused on system quality and user satisfaction. Most systems were evaluated at a feasibility stage or during small-scale pilot testing. Although the results of the evaluations were generally positive, biases in the methodology of evaluation were concerning selection, performance and exclusion. Gold standards and statistical tests were not always used when assessing diagnostic validity and patient management.

**Conclusions:**

Image-based telemedicine systems for injury emergency care tend to support valid diagnosis and influence patient management. The evidence relates to a few clinical fields, and has substantial methodological shortcomings. As in the case of telemedicine in general, user and system quality aspects are poorly documented, both of which affect scale up of such programs.

## Introduction

Rapid advances in telecommunication and information technology have sparked the development of a variety of systems that allow for new forms and domains of medical consultation. During the past two decades, many broad reviews of telemedicine have been published, describing the state of knowledge and assessing – to some extent – the quality of the evidence at hand. Some reviews are wide ranging both in scope and geography [1], [2], some are broad in scope but restricted to some countries [3], some deal with specific perspectives of application (like diagnostic and management decisions) [4], [5], and rare ones look at costs [6]. Two recent systematic reviews added to the literature in this area: one assessed the effect of telemedicine on professional practice and on patient health care outcome [7] and the other was a systematic review of reviews about the effectiveness of telemedicine [8]. A consistent finding across reviews is that radiology, mental health, and dermatology are three domains of application with positive clinical outcomes [4], [5]. Yet, there are serious concerns that the evaluations conducted thus far are of rather poor methodological quality (e.g., design, methods, size/dimension) [9], with weak theoretical foundations, and limited to assessments of clinical management rather than patient recovery (and health). Also, little is known regarding their sustainability or the manner in which they can be implemented in other settings [4], [5].

In the particular case of expert advice in the acute care of injured patients, expert consultation by telephone could be expected to significantly improve care access, quality and outcome by decentralising knowledge, speeding up and improving decision making and limiting patient transfer or expert displacement. This is encouraging as injury is an increasing cause of concern worldwide and it affects people from resource poor areas – where prognosis is not so good - to a far greater extent [10], [11]. Reviews are available in this domain, but many are descriptive [12]–[14] or context specific [4], [15]. A 2006 review focusing on accident and emergency telemedicine for primary care concluded that most studies conducted until then demonstrated technical feasibility and improved triage with an increasing range of local management, but few cost-effectiveness assessments were available [16]. Those reviews briefly introduced the role of telemedicine in the emergency department [14], current trends in the development and adoption of tele-medical adjuncts for injury control [17], potential applications/functions of telemedicine for trauma and disaster management, and a review of systems from a US perspective [15]. Successful domains of application identified thus far are the transmission of computed tomography scans for urgent neurosurgical opinion and the transmission and interpretation of radiographs (usually peripheral limb films) for on going support of minor injury units [12], [13]. In the case of burn injuries, studies are consistent on technical and clinical feasibility whereas less is known as regards clinical outcomes [18]. Systems have been evaluated in the main for their clinical accuracy, health care provider satisfaction, and follow-up of wound care [15].

Consulting those reviews helps us understand where the knowledge stands and what ethical and legal challenges are posed by the use of telemedicine in acute care. The knowledge at hand informs about various aspects of telemedicine, including those where experts are consulted and/or involved remotely in patient care. Yet, they provide limited assessments of the quality of the evidence thus far and they mix various types of telemedicine without specifying whether their conclusions actually apply to all of them. Although the field changes rapidly and new forms of teleconsultation enter the field of trauma care, those newer forms have not been reviewed in their own rights.

Against this background, this systematic review was undertaken to 1. revisit and update the literature specifically on image-based telemedicine for medical expert consultation in acute care of injuries; and 2. systematically review the evidence at hand regarding system, user and clinical perspectives. Four main research questions are addressed: What is the system quality? What is the diagnostic validity? What is the effect on the management and clinical outcomes? What is the level of user satisfaction?

## Methods

The procedures used in this review followed the PRISMA (Preferred Reporting Items for Systematic reviews and Meta-Analyses) Statement [19]. There is no published protocol for the systematic review, but the procedure is described in detail below.

The DeLone and McLean Information System Success Model [20] was used as a framework to synthesise the results according to system quality, user satisfaction, information quality (diagnostic validity) and net benefits (management and clinical outcomes).

### Date sources and searches

**Figure 1:**
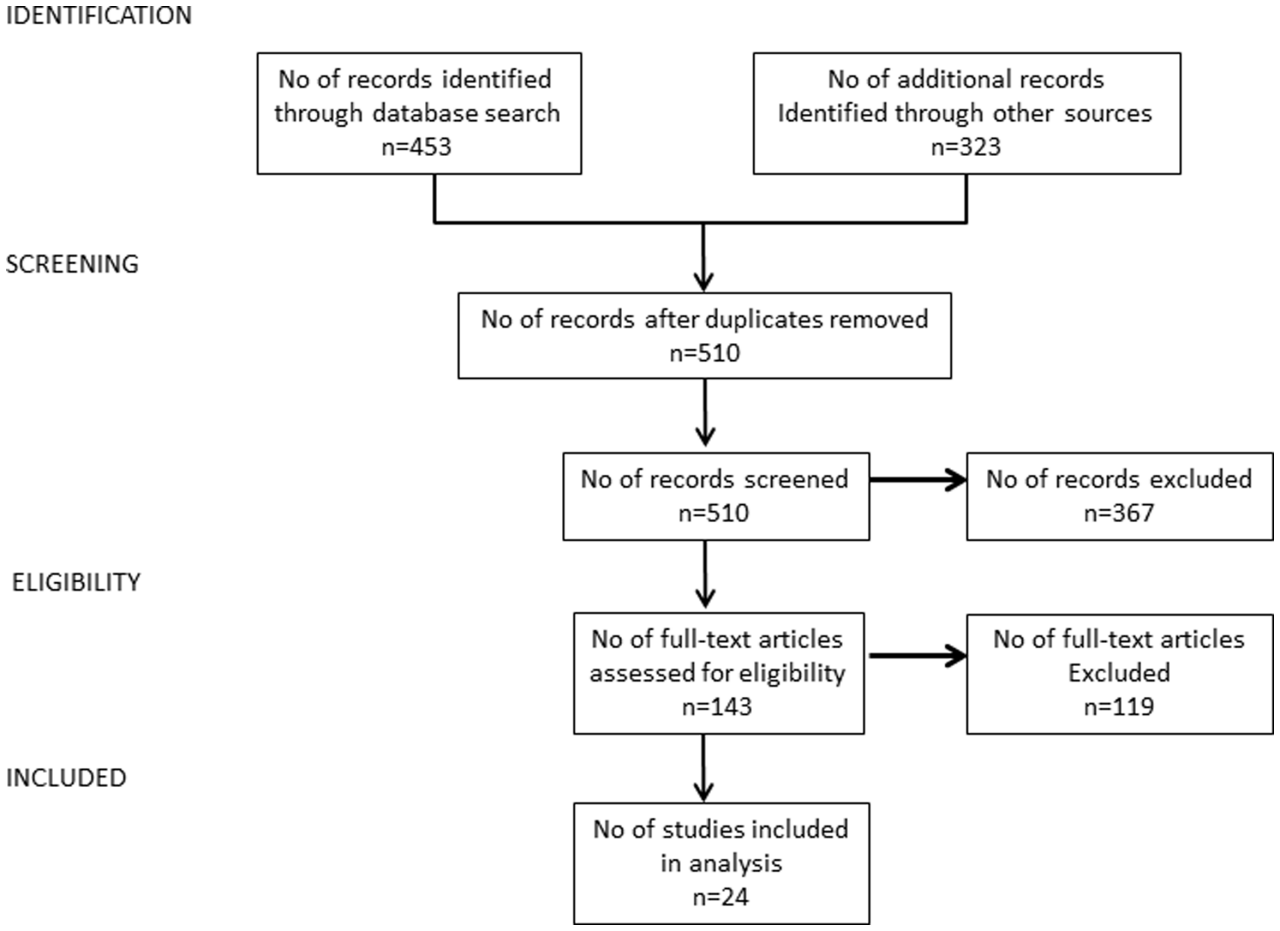
Flow chart of the literature search and screening process.

This systematic review includes studies that were published in articles from peer-reviewed journals. A systematic search identified potentially relevant articles in five electronic databases commonly used in this research area: MEDLINE, EMBASE, CINAHL, Cochrane Library and PsychINFO. The databases were searched without a time limitation in June 2012, with the following terms (in title and abstracts or as MeSH terms): “telemedicine”, “mHealth”, “m-health”, “eHealth”, “e-health”, “mobile health”, “emergency”, “emergencies”,” injury”, “injuries”, “trauma”, "acute burn", "acute burns". Relevant articles were also sought from the list of references of the reviews identified in the search, all articles from the archive of all online issues of the “Journal of Telemedicine and Telecare” and “Telemedicine and e-Health” Journal, starting from 2005 and from the list of references of the articles considered as eligible (see Figure 1 below).

### Study selection

Articles on the subject of telemedicine for medical expert consultation in emergency care were included if they met the following criteria: evaluated the acute stage of injury/trauma care, in emergency or pre-hospital settings; telemedicine intended to be used from point of care to specialist, and including image transfer; the system was assessed using human subjects; articles written in the English, French, Spanish, German or Nordic languages.

Studies were excluded due to the following criteria: reviews, case studies or purely descriptive studies; done under extreme conditions (disaster situations, war zones, space, etc.); image transferred does not consist of trauma images, and if image transfer was done in conjunction with real-time interactive video-conferencing.

First, two reviewers independently evaluated the abstracts of all records identified in the initial databases search. If either reviewer could not exclude the article, the full-text was obtained and evaluated by the reviewers to assess eligibility. Secondly, additional references from reviews, journals and eligible articles were screened by title by one of the reviewers. If the title indicated relevance, the abstract and then the full-text were screened by two reviewers.

### Data extraction and quality assessment

The articles were reviewed by all authors and after that a decision were made regarding the items that could be measured across most studies. Data was extracted on country, type of image, and the clinical focus that included the medical discipline and required expert, and the technology used for image treatment. From the four perspectives investigated, i.e. system quality, user satisfaction, diagnostic validity and clinical management, system quality and user satisfaction were seldom evaluated and the data gathered on those aspects related to the following. S*ystem quality* considered above all on image quality, and time to complete different steps in the telemedicine process and u*ser satisfaction* included the perceived ease of use and usefulness of the system. In the case of *diagnostic validity* and *management outcomes* a wider range of data were compiled relative to the methodology of the studies, including e.g., sample size and statistics used, and to the results obtained.

Attention was also paid to the methodological rigor of the studies by considering how various potential sources of bias were dealt with, based on “The Cochrane Collaboration's tool for assessing risk of bias” [21]: selection, performance, detection and attrition. The use of a “gold standard” was an additional criteria used for the studies dealing with diagnostic validity and management outcomes.

Data extraction for each article involved at least two reviewers who independently reviewed each article. After the individual assessments, the reviewers met two by two to discuss and agree on the data extraction and quality assessment of each article.

## Results

Of the 331 articles identified in the database search, 16 were eligible for review, an additional 3 were obtained from screening of relevant references from reviews, and 5 were obtained from screening the references of the eligible articles (Figure 1). The 24 articles describe 22 telemedicine systems, with two articles by Hsieh [22], [23] describing one system, and two articles by Wallace [24], [25] describing another.

The articles were published between 1992 and 2011, and were mostly carried out in high-income countries. They appeared in 18 different journals, two of which were from the telemedicine field and the others from medical journals (Table 1).

**Table 1:** Eligible articles by year of publication, country and journal type.

Country	Journal type	19								20											
		92	93	94	95	96	97	98	99	00	01	02	03	04	05	06	07	08	09	10	11
USA	Medicine(n = 9)	[38]	[30]		[33]		[37]	[28]	[29]			[43] [32]	[36]								
	Telemedicine(n = 2)									[27]										[44]	
UK	Medicine(n = 5)											[26]		[41]	[31]			[25]	[40]		
	Telemedicine(n = 2)																[24]				[45]
Europe(Other countries)	Medicine(n = 2)													[35]						[34]	
	Telemedicine (n = 0)																				
Asia	Medicine(n = 3)						[42]							[22]	[23]						
	Tele-medicine(n = 1)																[39]				

Table 2 describes some general characteristics of the systems investigated, ordered according to their stage of development: feasibility studies; pilot or small-scale roll-out studies; and post-implementation studies. These characteristics include the conditions assessed, which belonged to different medical disciplines and mostly general traumas, followed by orthopaedic and hand injuries, that most often required the expertise of plastic surgeons, followed by radiologists and orthopaedic surgeons. Images were captured, transmitted and displayed through various technologies, and there were two types of images transmitted: radiological images and clinical photographs of the injury. As also shown in Table 2, most articles reported on management outcomes and diagnostic validity (perspective), while others assessed user satisfaction and system quality.

**Table 2:** Description of eligible articles regarding origin, application and condition, image treatment and perspectives assessed.

Article	Country	Image type and clinical focus	Image treatment	Perspective			
				S	U	D	M
**Preparatory/feasibility studies**							
Mair 2011 [45]	UK	**Image:** Radiological**Discipline:**General injury**Experts:** Emergency physicians	**Capture:** Picture Archiving and Communications System (PACS) Document camera AV-P750, JVC**Transmission:** ISDN**Display:** A projected XGA image (low resolution form of PACS)				X
Egol 2003 [36]	USA	**Image:** Radiological and clinical**Discipline:** Orthopaedic**Experts:** Orthopaedic surgeons	**Capture:** Radiographs scanned via a telemedicine scanning system**Transmission:** Not applicable**Display:** Not described		X		X
Jacobs 2002 [26]	UK	**Image:** Radiological**Discipline:** General injury**Experts:**Oral and maxillofacial surgeons	**Capture:** Remote Video Expertise™ RVE version 1.0.11.0**Transmission:** 6 ISDN telephone lines**Display:** Computer	X		X	
Krupinski 2000 [27]	USA	**Image:** Radiological**Discipline:** General trauma**Experts:** Orthopaedic surgeons and radiologists	**Capture:** Digital camera**Transmission:** Private asynchronous transfer mode network based on T1 carriers**Display:** Colour monitor	X		X	
Raikin 1999 [29]	USA	**Image:** Radiological**Discipline:** Orthopaedic**Experts:** Orthopaedic surgeons	**Capture:** Scan (Scanjet 4sc scanner) saved as JPEG files**Transmission:** email**Display:** Computer	X		X	X
Larson 1997 [28]	USA	**Image:** Radiological**Discipline:**Spinal**Experts:** Radiologists	**Capture:** Scan (Lumiscan 150 scanner)**Transmission:** Not described**Display:** Monitor of an Agfa review station.	X		X	
Reid 1997 [37]	USA	**Image:** Radiological**Discipline:** Orthopaedic**Experts:** Orthopaedists	**Capture:** Pan-tilt-zoom camera and converted to digital format.**Transmission:** Triple ISDN**Display:** Colour monitor			X	
Wilson 1995 [33]	USA	**Image:** Radiological**Discipline:** General trauma**Experts:** Radiologists	**Capture:** Laser digitizer Kodak FD-1S**Transmission:** Ethernet link**Display:** Kodak PDS-2 Workstation with monochrome display monitors.	X		X	
Scott 1993 [30]	USA	**Image:** Radiological**Discipline:** Orthopaedic**Experts:**Radiologists	**Capture:** Digitized with a laser digitizer**Transmission:** Not applicable**Display:** Viewing station monitors	X		X	
Yoshino 1992 [38]	USA	**Image:** Radiological**Discipline:**Spinal**Experts:** Neuroradiologists	**Capture:** Digitizer**Transmission:** Dedicated telephone line**Display:** Images rewritten onto a single emulsion film using an LR1 laser printer			X	
**Pilot/small scale roll-out studies**							
Abou Al Tout 2010 [34]	France	**Image:**Clinical**Discipline:**Hands**Experts:**Hand surgeons	**Capture:** Digital camera with video function**Transmission:** Internet**Display:** Computer	X			X
Diver 2009 [40]	UK	**Image:**Clinical and radiological**Discipline:**Hands**Experts:**Plastic surgeons	**Capture:** Digital camera**Transmission:** Not described**Display:** Computer			X	X
Chandhanayingyong 2007 [39]	Thailand	**Image:** Radiological**Discipline:** General injury**Experts:**Not specified	**Capture:** Mobile phone camera**Transmission:** MMS**Display:** Camera phone			X	X
Archbold 2005 [31]	UK	**Image:** Radiological**Discipline:** Orthopaedics**Experts:**Trauma surgeons	**Capture:** Mobile phone camera**Transmission:** MMS**Display:** Mobile phone	X	X	X	X
Hsieh 2005 [23]	Taiwan	**Image:**Clinical and radiological**Discipline:**Hands**Experts:**Plastic surgeons	**Capture:** Mobile phone camera**Transmission:** Sent to a mobile phone or as an email**Display:** Mobile phone or computer	X		X	
Hsieh 2004 [22]	Taiwan	**Image:**Clinical and radiological**Discipline:**Hands**Experts:**Plastic surgeons	**Capture:** Mobile phone camera**Transmission:** Global system for mobile communication over the tri-band frequency**Display:** Mobile phone	X		X	X
Poca 2004 [35]	Spain	**Image:** Radiological**Discipline:**Head**Experts:** Neurosurgeons	**Capture:** Digitalized CT scans**Transmission:** Internet**Display:** Computer	X		X	X
Jones 2004 [41]	UK	**Image:**Clinical and radiological**Discipline:** General trauma**Experts:**Plastic surgeons	**Capture:** Digital camera**Transmission:** email via intranet**Display:** monitor screen			X	X
Pap 2002 [32]	USA	**Image:**Clinical and radiological**Discipline:** Plastic surgery**Experts:** Plastic surgeons	**Capture:** Digital camera**Transmission:** email**Display:** Not described	X	X	X	X
Ricci 2002 [43]	USA	**Image:** Radiological**Discipline:** General trauma**Experts:** Orthopaedic surgeons	**Capture:** Digital camera**Transmission:** Ethernet connection (when in the hospital network), email or dial-up networking**Display:** Computer				X
**Post-implementation (implemented system)**							
Moya 2010 [44]	USA	**Image:** Radiological**Discipline:**Head**Experts:**Neurosurgeons	**Capture:** Digital images (CT, MRI, ultrasounds, X-ray)**Transmission:** Internet**Display:** Computer				X
Wallace 2008 [25]	UK	**Image:**Clinical and radiological**Discipline:**Plastic surgery**Experts:**Plastic and maxillofacial experts	**Capture:** Not described**Transmission:** email via intranet**Display:** Computer		X		X
Wallace2007 [24]	UK	**Image:**Clinical and radiological**Discipline:** Plastic surgery**Experts:**Plastic and maxillofacial experts	**Capture:** Digital camera**Transmission:** email**Display:** Not described		X		X
Goh 1997 [42]	Hong Kong	**Image:** Radiological**Discipline:**Head**Experts:** Neurosurgeons	**Capture:** CT scans**Transmission:** Telephone lines**Display:** Computer			X	X

Perspectives: S = system quality, U = user satisfaction, D = diagnostic validity, M = management outcomes.

**Figure 2:**
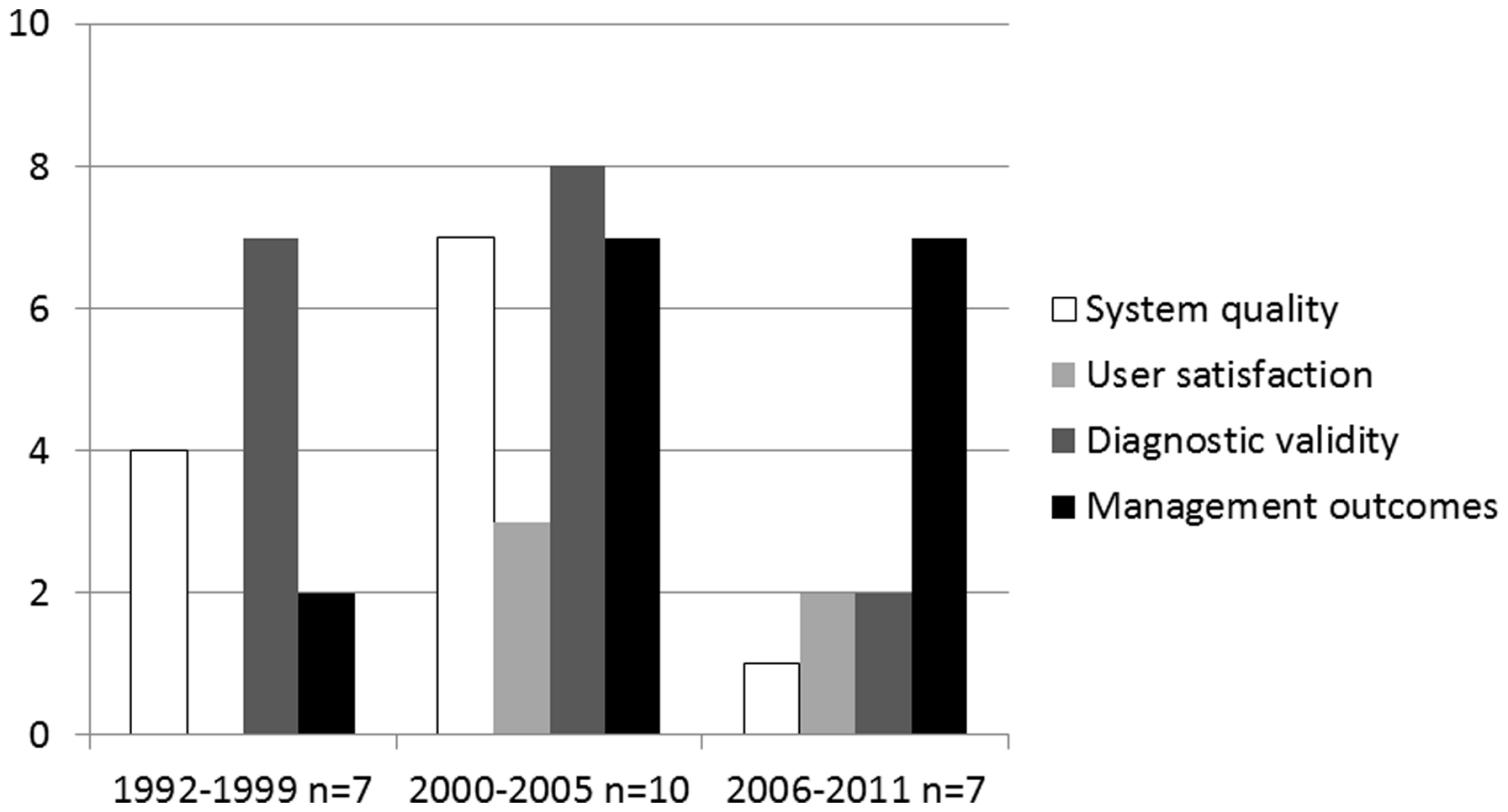
Perspectives of articles by year of publication.

Figure 2 represents the number of articles that report on different perspectives, within three different time periods. There is a trend whereby more recent articles seem to be focusing more on management outcomes and less on diagnostic validity.

### System quality

12 articles assessed whether image transfer provides adequate support for injury acute care assisted by telemedicine. These articles evaluated the quality of the images [22], [26]–[32] and how long it takes to complete different steps in the telemedicine process [22], [23], [32]–[35]. In some cases assessment of image quality was done using scales, and in others it was not clear how the assessments were made. Image quality was considered lower for telemedicine compared with original radiographs in a few of the studies [26]–[28], [30]. Users in other studies expressed satisfaction with the telemedicine image quality [22], [29]–[32]. In one of the studies, the quality of the telemedicine images was rated lower than the original radiographs, although the users were still satisfied with those images[30]. Operation time (from taking the image to reception of the image) was 3 to nearly 15 minutes [22], [23], [32] and the time for creating a file was 3 to7 minutes [34]. One study indicated that telemedicine radiographs took longer time to read than originals [33], and another one that telemedicine increased the time at the emergency department [35].

### User satisfaction

Five articles report user data [24], [25], [31], [32], [36]. They address the ease of use [24], [25], [31] and the perceived usefulness of the system for clinical decision making [24], [25], [32], [36]. Three indicate that the data were gathered by questionnaire, [24], [31], [36] but none specifies how the question read, what the alternative answers were, and whether the questions – or answers – were standardized or validated. Overall, the studies report high levels of satisfaction and perceived ease of use.

### Diagnostic validity

Table 3 presents the 17 articles that assessed diagnostic validity. Eight assessed systems at the feasibility stage, and nine at the pilot and post-implementation stages. The former all used radiological images, and had different designs whereby the assessors would either assess the images by one modality (i.e. either the original or digitized radiograph)[30], [37], digitized images before the original ones [27]–[29], original images before the digitized ones [26], or mixed modalities where some assessors started with original and others started with digitized radiographs [33], [38]. In one article [29] the description of the radiograph was also assessed and compared to the digitized and original radiographs. Assessments by both modalities were done one directly after the other [28], [29], two weeks apart [26], at least four weeks apart [33], [38], or six months apart [27]. All studies used a gold standard, and employed accuracy (sensitivity and specificity) measurements, Receiver Operating Characteristic (ROC), Kappa or McNamar's test.

**Table 3:** Diagnostic validity.

Article	Image type/discipline	Sample size	Methodology	Results	Methodological limitations
**Preparatory/Feasibility studies**					
Jacobs et al. 2002 [26]	Radiology/General injury	Images: n = 2010 orthopantomographs (OPGs) (5 with and 5 without fractures),10 occipitomentals (OMs) (5 with and 5 without fractures)Assessors: n = 168 Oral and maxillofacial surgeons (OMFS) and 8 accident and emergency (A&E) doctors	**Procedure:**Original radiographs viewed by OMFS and A&E doctors.Telemedicine images viewed by OMFS 2 weeks after assessing the originals.**Outcome:**Diagnostic accuracy**Instrument:**Questionnaire completed for each radiograph viewed.Confidence scores of diagnosis on a scale of 1–10.**Statistics:**Sensitivity and specificity in fracture diagnosis and identification of the position (overall and split into 10 highest and 10 lowest quality radiographs)**Gold standard:**The assessment panel's assessment on the original radiographs.	**Fracture diagnosis:**Sensitivity: Original radiographs OMFS: 100/A&E: 90; Telemedicine OMFS: 86Specificity: Original radiographs OMFS: 84/A&E: 77; Telemedicine OMFS: 8010 high quality radiographs - sensitivity and specificity of OMFS with telemedicine higher than A&E doctors with original radiographs.10 low quality radiographs, sensitivity and specificity of OMFS with telemedicine lower than A&E with original radiographs.Poor quality radiographs and frontozygomatic and infraorbital rim fractures were poorly diagnosed by telemedicinePosition of the fracture more accurately assessed using original radiographs.Diagnosis by OMFS doctors using telemedicine was broadly comparable with fracture diagnosis by A&E doctors using original radiographs.Mean confidence scores: Original radiographs OMFS: 6.8/A&E: 7.1; Telemedicine OMFS: 4.7	- Selection: Not described- Performance: Original radiographs assessed before the telemedicine by the same assessor
Krupinski et al. 2000 [27]	Radiology/General trauma	Images: n = 40 films of bone trauma casesAssessors: n = 42 orthopaedic surgeons and 2 radiologists	**Procedure:**Each assessor viewed the digital images. Six months later, they reviewed the original film.**Outcome:**Diagnostic accuracy and confidence in diagnosis.**Instrument:**A 6 point scale from 1(no lesion present, definite) to 6 (lesion present, definite).**Statistics:**Receiver Operating Characteristic (ROC) analysis was performed on the confidence values.The Multi-Reader Multi-Case (MRMC) ROC analysis technique was used.Kappa: agreement between original film and digitized reading for each observer.**Gold standard:**Diagnoses by two radiologists who assessed the original films.	No significant difference in diagnostic accuracy between original film and digital image.No significant differences in performance among the 4 observers.Kappa values: 0.94 and 0.92 for the radiologists, 0.89 and 0.88 for the orthopaedists.43% of the confidence ratings were exactly the same for film and photo viewing. 53% differed by only one category. Major differences were most often those images that were judged as poor quality and/or had poor framing.48 of 320 decisions (15%) were incorrect.	- Selection: Convenience sampling, although with clearly described inclusion criteria- Exclusion: Images where the original film was of poor quality were not evaluated
Raikin et al. 1999 [29]	Radiology/Orthopaedic	Images: n = 25radiographsAssessor: n = 44 orthopaedic surgeons	**Procedure:**Assessors: 1) read a description of the radiographs, 2) viewed the telemedicine image, 3) viewed original radiographs.**Outcome:**Diagnosis decision**Instruments:**Questionnaire after each stage, regarding change in diagnosis.**Statistics:**Chi-square analysis**Gold standard:**Not specified	Overall, a significant improvement in the frequency of correct diagnosis and treatment planning when digitized images were used (91%) compared with textual descriptions alone (48%) (p<0.001).Correct diagnosis and classification- By the initial description of the injury: 48%; by digitized radiograph: 91%,; only 3 diagnosis changed after seeing the original radiograph.- Significant difference between verbal description and the other groups (p<0.001), but not between the digitized and original radiographs (p = 0.27).Significant differences between verbal descriptions and digitized radiographs in the surgeons' ability to appreciate:- Severity of injury: 34% vs. 97% (p<0.001)- Degree of comminution: 18% vs. 98% (p<0.001)- Degree of articular involvement: 23% vs. 93% (p<0.001)Where comminution and articular involvement could not be assessed, original films did not significantly add to the understanding.	- Selection: Unclear what the cases are representative of- Gold standard not described
Larson et al.1998 [28]	Radiology/Spinal	Images: n = 5529 with normal findings and 26 with subtle fracturesAssessors: n = 3radiologists	**Procedure:**Each assessor viewed the digitalized image and then the original image.**Outcomes:**Determination of presence of abnormalities**Instruments:**The confidence scale used the following values: 1 = definitely normal, 2 = probably normal, 3 = equivocal, 4 = probably abnormal. 5 = definitely abnormal.**Statistics:**For comparison of sensitivity and specificity, a McNemar test for paired proportions was used.ROC analysis**Gold standard:**Consensus of two board-certified radiologists	For subtle fractures, the sensitivity when using a teleradiology system was similar to that of conventional radiographs.Sensitivity: original radiograph: 88%; digitized radiograph: 87%Specificity: original radiograph: 79%; digitized radiograph: 83%All cases were detected by at leas one radiologist; 6 of 26 fractures were missed by at least one radiologist on the original and digitized images.ROC analysis showed that the differences between original and digitized images were not statistically significant for any of the three radiologists.	- Selection: Convenience sampling, although with clearly described inclusion criteria- Performance: Same assessor evaluated the image by both modalities one right after the other
Reid et al. 1997 [37]	Radiology/Orthopaedic	Images: n = 80Cases with various degrees of complexity.Assessors: n = 42 radiologists and 2 orthopaedists.	**Procedure:**Assessors reviewed either teleradiology or original radiographs**Outcome:**Diagnosis and relative certainty of the diagnosis**Instruments:**Certainty measured on a scale of 1-5Accuracy of diagnosis; dichotomized in precise or wrong diagnosis.**Statistics:**McNemar's test.**Gold standard:**The diagnosis of the attending orthopaedic surgeon who treated the case.	80% of the diagnosis of the telemedicine and original radiographs was concordant. A precise consensus diagnosis in 66% of the cases (78% for orthopaedists/55% for radiologists).Precise diagnosis:Orthopaedists: 93% of the original radiograph readings and 80% of the telemedicine readings (not significant difference).Radiologists: 70% of the original radiograph readings and 63% of the telemedicine readings (not sig difference).Statistically significant difference between orthopaedists and radiologists for reading original films, but not for telemedicine films.For those instances when the diagnosis was imprecise, the residents were aware of their Inability to make an accurate diagnosis.Significant relationship between diagnostic accuracy and certainty of diagnosis in orthopaedists reading radiographs via telemedicine.Confidence in diagnosis:Orthopaedists and radiologists had the same confidence in their diagnosis when reading original radiographs (p = 1.000), but differed significantly when reading via telemedicine (p = 0.039).Significant difference in certainty and accuracy between the two viewing modalities for both the orthopaedists and the radiologists.	- Selection: Sampling not described
Wilson et al.1995 [33]	Radiology/General trauma	Images: n = 180Radiographs of skeletal trauma patients.Assessors: n = 44 radiologists	**Procedure:**Each reader looked at one set of cases as original film and the other set of as digitized images. After at least 4 weeks, cases seen as digitalized images were viewed as original and vice versa.**Outcome:**Identification of fractures and dislocations.**Instruments:**A 6 point scale (1 = definitely normal structure, 6 = definitely abnormal).**Statistics:**ROC analysis for each reader and each reading method.Accuracy, sensitivity and specificity of dislocations.**Gold standard:**Clinical and radiologic follow-up from medical records or consensus opinion of the original readings, study radiologists readings and final assessment by authors.	Intra-rater significant difference between original films (superior) and digitized images for 3 of 4 radiologists.Total fractures – statistically significant differences between original film and digitized images.Subtle fractures – ROC curves showed superior performance for original film with only three readers and only one was statistically significant.Non-subtle fractures –all readers performed better on original film, but the differences were statically significant for only two radiologists.For dislocations, calculations of sensitivity, specificity and accuracy were not significantly different for any reader between the original and digitized images.	- Selection: Convenience sampling, although with clearly described inclusion criteria, from two sources
Scott et al. 1993 [30]	Radiology/Orthopaedic	Images: n = 12060 cases with fractures/dislocations60 controls with similar ageAssessors: n = 87 senior radiology residents and 1 fellow	**Procedure:**Each assessor viewed 60 random cases with the original film and then 60 other cases using teleradiology (they did not view the same case in different modalities).**Outcome:**Diagnosis and confidence.**Instruments:**Confidence ratings of low, moderate, or high and a positive or negative reading, which corresponded to 6 point scale from “almost definitely negative” to “almost definitely positive”.**Statistics:**ROC analysisFrequency, accuracy, specificity and sensitivity**Gold standard:**Interpretations by three authors. When three or more readers misinterpreted a case, a consensus panel was used.	Overall accuracy of the readers: 80.6% for original film interpretations and 59.6% for digitized readings (P<.001).Sensitivity: 78.5% for original film and 48.8% for digitized images (P<.001).Specificity: 83.2% for original film and 72.3% for digitized images (P<.025).Original film readers produced significantly better results (p<0.05) than digitized readings for four of the eight readers in accuracy and for five of the eight in sensitivity.No significant difference in specificity for any of the individual readers.After the data were pooled, original film readings produced significantly better results for all three measures (accuracy, sensitivity and specificity).Accuracy and sensitivity were significantly less for digitized images within each of the 3 image quality categories, and especially low in moderate and high difficulty cases in the digital mode.ROC analysis showed a significant difference between original and digitized images.	- Selection: Convenience sampling, although with clearly described inclusion criteria
Yoshino et al.1992 [38]	Radiology/Spinal	Images: n = 5025 radiographs of cervical spine fractures and 25 radiographs without fractures.1 radiograph per patient, selected by the author.Assessors: n = 42 neuroradiologists, 1 neuroradiology fellow, 1 general radiologist.	**Procedure:**Each assessor viewed the images using both modalities, with at least four weeks apart. Two began with original images and two began with telemedicine images.**Outcome:**Diagnostic accuracy**Instruments:**Level of certainty of fracture (1 = fracture definitely present to 6 = fracture definitely not present).Location of fracture**Statistics:**ROC analysis from each reader and each reading method.**Gold standard:**Fractures were proven by autopsy, surgical findings, tomography or follow-up examination.	2 of the 4 readers had statistically significantly (p = 0.05) better fracture detection using original radiograph.Pooled ROC scores for all readers were 0.904 for original radiographs and 0.868 for telemedicine images.	- Selection: Convenience sampling
**Pilot/small scale roll-out studies**					
Diver et al. 2009 [40]	Clinical image and radiology/Hands	Images: n = 20From trauma patientsAssessors: n = 1Plastic surgery registrar	**Procedure:**Each patient assessed at a trauma clinic by a house officer (to mirror an AED doctor). The registrar viewed the image in combination with telephone contact, and then the patients assessed the patients face-to-face.**Outcome:**Discrepancy in diagnosis**Instrument:**Not mentioned**Statistics:**No**Gold standard:**The registrar assessed each patient in person. However, this was not used as gold standard in a statistical test.	In 1 of 20 cases the face-to-face consultation highlighted patient history details that were not obtained through the consultation.In 1 of 20 cases, a discrepancy in examination findings was identified between the face-to-face examination and the transmitted image.	- Selection: Not described- Performance: Same assessor evaluated the image by both modalities one right after the other- No statistical tests- No use of a gold standard
Chandhanayingyoung et al. 2007 [39]	Radiology/General injury	Images: n = 720From 93 patients (59 emergency orthopaedic patients diagnosed with a non- or minimally displaced fractures and 34 age-matched normal patients)Assessors: n = 42 senior staff and 2 junior staff	**Procedure:**Each assessor conducted two evaluations of the digital images, with two weeks in between.**Outcome:**Determining the presence of fracture and location of fracture.**Instrument:**Data collection form**Statistics:**Kappa statistic was used to test for level of inter and intra-observer agreements.Sensitivity, specificity and accuracy of each group of assessors.Chi-squared to test the association between variables and misdiagnosis.**Gold standard:**Clinical and radiographic follow up data. When not available, a panel of 3 specialists.	Both inter and intra-observer agreement were good (kappa<0.60):Inter-rater agreement: kappa = 0.67 (good)Intra-rater agreement: kappa = 0.68 (good)Overall sensitivity was 78% at 1^st^ assessment and 80% at 2^nd^ assessmentOverall specificity was 57% at 1^st^ assessment and 54% at 2^nd^ assessmentOverall accuracy was 66% at 1^st^ assessment and 65% at 2^nd^ assessment.Misdiagnosis:- Overall misdiagnosis rate: 40%. 12% over-diagnosis, 27% under-diagnosis.- No association was found between the experience of the assessors, the region of the fracture or the age group of the patients and the misdiagnosis rate.	- Authors state the limitation of having more than one source for the gold standard
Archbold et al. 2005 [31]	Radiology/Orthopaedics	Images of 46 consultationsAssessors: n = not mentionedTrauma surgeonsand referring emergency physicians	**Procedure:**1) Assessment after telephone referrals2) Assessment after multi-media consultations**Outcome:**Accuracy of injury description**Instrument:**Not mentioned	In 10 cases the MMS revealed that the initial description of the injury was inaccurate with respect to the actual injury.	- No statistical tests- No use of a gold standard
Hsieh et al.2005 [23]	Clinical image and radiology/Hands	Images: n = 12835 patients with 60 digit injuriesAssessors: n = 3plastic surgeons	**Procedure:**The assessors reviewed image together with a brief patient history.**Outcome:**- Injury extent- Ability to identify the location of amputation- Status of amputation level- Presence of distal ecchymosed skin along the digital arteries.**Instruments:**A standard questionnaire.**Statistics:**Sensitivity and specificity of remote diagnosis of distal skin ecchymosis and replantation potential - calculated when all 3 surgeons agreed.**Gold standard:**On-site evaluation by the consultant attending plastic surgeon	Identified by all 3 surgeons:- Amputation location in 90% of the 60 digits.- Status of amputation level In 87% of the 60 digits.- Recognition of the presence of distal skin ecchymosis along the digital artery: 79% sensitive and 90% specific.- Recognizing digital replantation potential was 90% sensitive and 83% specific.	- Selection: Convenience sampling, although with consideration of severity level- Performance: Authors participate as assessors
Hsieh et al. 2004 [22]	Clinical image and radiology/Hands	Images: n = 18445 patients with 81 digital injuriesAssessors: n = 3junior plastic surgery residents	**Procedure:**The assessors reviewed image together with a brief patient history.**Outcome:**Identification of extent of injury (skin defect or bone exposure)**Instruments:**A standard wound questionnaire**Statistics:**Sensitivity and Specificity of remote diagnosis of wound descriptors (skin defect or bone exposure) were calculated under group agreement.**Gold standard:**Consultant surgeon viewed all patients in the emergency room shortly after the initial telemedicine referral.	Remote diagnosis of the skin defect: 79% sensitivity and 71% specificity.Remote diagnosis of bone exposure: 76% sensitivity and 75% specificity.	- Selection: Not described- Performance: Authors participate as assessors
Poca et al. 2004 [35]	Radiology/Head	Images: n = 90 teleradiological examinationsAssessors: n = not mentionedA neuroradiologist and the neurosurgeon on call.	**Procedure:** 90 images were evaluated by the neuroradiologist and neurosurgeon on call independently.**Outcome:**Discrepancy between the neuroradiologist and the neurosurgeon on call.**Instrument:**Not mentioned	Of the 90 cases reviewed by both assessors, the neuroradiologist detected 4 mild injuries that were not detected by the neurosurgeon on call.	- No statistical tests- No use of a gold standard
Jones et al. 2004 [41]	Clinical image and radiology/General trauma	Images: n = 82Assessors: n = Not mentionedTrauma team: Senior House Officer (SHO), registrar, consultant.	**Procedure:**Cases were assessed by reviewing the telemedicine images together with a conventional telephone referral, and re-assessed on arrival to the minor injury unit.**Outcomes:**severity (grade of injury)**Instruments:**A five-point scale, devised by the authors.**Statistics:**Correlation coefficient.**Gold standard:**Patient re-assessed on arrival. However, this was not used as gold standard in a statistical test.	Accuracy of transmitted image in comparison to injury on examination was >97%.All surgeons had closely matched scores for grade of injury.Overall, consultant achieved the highest correlation coefficient when compared to the more junior members of the team.	- Selection: Not described- Performance: Assessments in the two modalities may have been done by the same team- Exclusion: Some images were not evaluated- inadequate or lost data- No use of a gold standard
Pap et al. 2002 [32]	Clinical image and radiology/Plastic surgery	Images: n = 20Assessors: n = 4Attending plastic surgeons	**Procedure:**The assessors reviewed the digital images together with a telephone call.**Outcome:**Clinical description.**Instruments:**Not mentioned	The clinical descriptions were clear and the diagnoses precise in all instances.	- Selection: Convenience sampling, although in random order- No statistical tests- No use of a gold standard
**Post-implementation (Implemented system)**					
Goh et al.1997 [42]	Radiology/Head	Images: n = 3128 patients referred by telephone; 35 patients referred with teleradiology images.Assessors: n = not mentionedNeurosurgeons	**Procedure:**Neurosurgeons reviewed cases either by telephone consultation or by telephone consultation together with transmitted images.**Outcome:**Diagnostic accuracy - agreement between referring doctor and neurosurgeon.**Instruments:**Not mentioned**Statistics:**Fisher's exact	There was generally good agreement in CT diagnosis between the referring doctor and neurosurgical team.Only one case where the referring doctor missed a condition that had no impact on patient management in the acute phase.	- No use of a gold standard

In five of the nine other studies that assessed systems at the pilot or post-implementation stages, the assessors considered cases through the telemedicine modality only [22], [23], [32], [35], [39]; in two, both telemedicine and on-site interpretations were done one after the other [40], [41]. In two articles a telephone description was compared to the telemedicine modality [31], [42]. Three evaluations used a gold standard [22], [23], [39], and statistical analysis included kappa, correlation coefficient and descriptive statistics, and accuracy analysis (sensitivity and specificity). Results showed generally good diagnostic accuracy, except in one study [39].

The main limitation of the evidence at hand was that 7 of the studies did not specify a gold standard [29], [31], [32], [35], [40]–[42] and that four studies did not use statistical tests to validate the diagnosis [31], [32], [35], [40]. Convenience sampling were often used, in some studies clearly described [23], [27], [28], [30], [32], [33], [38], but in others not [22], [26], [29], [37], [40], [41]. Even if this limits the general representativeness of the studies, it may reflect specific or complicated diagnosis. Some of the articles did not clarify the performance of the studies [22], [23], [26], [28], [40], [41] which made it difficult to review the rigor of those studies.

### Management and clinical outcomes

Table 4 presents the 16 articles that assess the effect of image-based telemedicine on the clinical management of patients. In these articles, management plans after viewing digitized images were compared with written or oral descriptions [24], [25], [29], [31], [36], [42]–[44], original radiographs or on-site examination [29], [40], [41], [43], and video [45]. Others estimated the consequence of misdiagnosis [39], or compared to the management suggested by the referring doctor [32].

**Table 4:** Management outcomes assessment.

Article	Image type/discipline	Sample size	Methodology	Results	Methodological limitations
**Preparatory/Feasibility studies**					
Mair et al. 2011 [45]	Radiology/General injury	Images: n = 33Assessors: n = 20Emergency physicians	**Procedure:**60 case reviews were conducted by video link or telephone call with viewing of digital images (PACS), in five sessions, held approximately four weeks apart. Some cases were presented by both modalities.**Outcome:**- A working management plan- Confidence in making the working management plan- Locally treated or transfer**Instrument:**Not mentioned**Statistics:**Kappa statistic was used to estimate within-observer agreement.Logistic regression (odds ratio)	Proportion of patients transferred was higher with PACS than video in 10 cases, lower in 5 cases and the same in 6 cases. Proportion of patients transferred was higher when PACS was used for all except 5/20 doctors.The estimated odds for patient transfer were 56% lower when video was used instead of PACS (OR = 0.44 95% CI 0.20–0.93).The estimated odds for patient transfer were 58% lower when a more experienced doctor was used instead of a less experienced one (OR = 0.42 95% CI 0.17–1.02)Intra-agreement about transfer between 2 reviews by the same modality and doctor was 82%, which resulted in a kappa statistic of 0.54.	- No use of a gold standard
Egol et al. 2003 [36]	Radiology and clinical image/Orthopaedic	Images from 11 orthopaedic emergency room consultationsAssessors: n = 50Voluntary physicians at a conference	**Procedure:**Assessments were made after clinical reading by the emergency room attending physician and after digitized images were shown.**Outcome:**Initial patient management in terms of:- admitting the patient- requiring surgery- coming to evaluate the patient- needing more information**Instrument:**Questionnaire before and after viewing the images	The majority did not change their answers regarding the initial treatment with the added information provided by telemedicine.- Admitting the patient: 83% remained unchanged.- Operative treatment: 78% remained unchanged.- Need of more info prior to making a clinical decision: 70% remained unchanged.Of 537 assessments, respondents agreed with the emergency room physician's interpretation in 264 instances (49%).	- Selection: Convenience sampling- Performance: Authors mention the difficulty of viewing in a large auditorium setting.- No statistical tests- No use of a gold standard
Raikin et al. 1999[29]	Radiology/Orthopaedic	Images: n = 25radiographsAssessors: n = 44 orthopaedic surgeons	**Procedure:**Assessors: 1) read a description of the radiographs, 2) viewed the telemedicine image, 3) viewed original radiographs.**Outcome:**Treatment decision**Instrument:**Questionnaire**Statistics:**Chi-square analysis	The difference in correct treatment plans between digitized images and actual radiographs was not significant (p = 0.27).It was possible to make a treatment plan, including need and type of surgery in 25% of the cases after verbal description. Treatment plan changed in 74% of the cases the decision to perform surgery and in 80% of the cases type of surgery planned would change, after seeing the digital image. An additional 5% would change after viewing the original radiograph.	- Selection: Unclear what the cases are representative of- No use of a gold standard
**Pilot/small scale roll-out studies**					
Abou Al Tout et al. 2010 [34]	Clinical image/Hands	Images: n = 460 (and 4 videos), from 129 patientsAssessors: n = 8:7 emergency physicians1 hand surgeon	**Procedure:**The emergency physicians reviewed the patients and the teleexpert reviewed the transmitted images.**Outcome:**Change in management/Observation**Instrument:**Not mentioned	In 19 cases, the management changed due to the consultation.4 times to modify medical prescription, 10 times to modify an orthopaedic or surgical procedure, 5 times to modify referral of the patient.	- Selection: Convenience sampling- No statistical tests- No use of a gold standard
Diver et al. 2009 [40]	Clinical image and radiology/Hands	Images: n = 20From trauma patientsAssessors: n = 1Plastic surgery registrar	**Procedure:**Each patient assessed at a trauma clinic by a house officer (to mirror an AED doctor). The Image was transmitted to the registrar in combination with telephone contact**Outcome:**Differences between telemedicine and face-to-face management decisions**Instrument:**Not mentioned	In 1 of 20 cases there was a difference between the management plan based on history/image analysis and the plan following face-to-face consultation.5 of 20 patients could have been adequately managed in a casualty department, thus Image analysis could have precluded the need for transfer.	- Selection: Not described- Performance: Same assessor evaluated the image by both modalities one right after the other- No statistical tests- No use of a gold standard
Chandhanayingyoung et al. 2007[39]	Radiology/General injury	Images: n = 720From 93 patients (59 emergency orthopaedic patients diagnosed with a non- or minimally displaced fractures and 34 age-matched normal patients)Assessors: n = 42 senior staff and 2 junior staff	**Procedure:**Each assessor conducted two evaluations of the digital images, with two weeks in between.**Outcome:**Estimated consequences of misdiagnosis**Instrument:**Data collection form	Consequences of misdiagnosis:- Would have resulted in mismanagement in up to 48% of the cases: Under treatment in up to 45% of adult cases and 29% in paediatric cases.	- No statistical tests- No use of a gold standard.
Archbold et al. 2005 [31]	Radiology/Orthopaedics	Images of 46 consultationsAssessors: n = not mentionedTrauma surgeonsand referring emergency physicians	**Procedure:**1) Assessment after telephone referrals2) Assessment after multi-media consultations**Outcome:**Effect on patient management**Instrument:**Questionnaire	MMS consultation was felt to have changed the initial management of the patients in 8/46 referrals.Feeling the MMS consultations improved the patient care: 34/46 cases among trauma surgeons and 36/46 cases among emergency physicians.	- No statistical tests- No use of a gold standard
Hsieh et al. 2004 [22]	Clinical image and radiology/Hands	Images: n = 18445 patients with 81 digital injuriesAssessors: n = 43 junior plastic surgery residents1 consultant plastic surgeon	**Procedure:**The consultant and residents reviewed image together with a brief patient history.**Outcome:**Triaging during remote consultation and actual treatment according to on-site inspection**Instrument:**Triage into 3 groups according to severity and management plan:Group I-conservative treatment.Group II-skin grafting or local flap coverage.Group III-microsurgery such as replantation or free flap coverage.	15% of cases with disagreement of triaging between the teleconsultation and the actual treatment by the attending surgeon.25% of cases with significant discordance among residents; difference partly attributable to the inability to show instances of tiny exposed digital bone or tendon in some cases.15% with residents' agreement regarding the triaging had a clinically significant misinterpretation of an image.	- Selection: Not described- Performance: Authors participate as assessors- No statistical tests- No use of a gold standard
Jones et al. 2004[41]	Clinical image and radiology/General trauma	Images: n = 82150 trauma referralsAssessors: n = Not mentionedTrauma team: Senior House Officer (SHO), registrar, consultant.	**Procedure:**Cases were assessed by reviewing the telemedicine images together with a conventional telephone referral, and re-assessed on arrival to the minor injury unit.**Outcome:**Operative priority.**Instrument:**A five-point scale, devised by the authors.**Statistics:**Correlation coefficient.	All surgeons had closely matched scores operative priority.The highest correlation was seen in scoring the operative priority of patient injuries (as compared to injury severity)Overall, consultant achieved the highest correlation coefficient when compared to the more junior members of the team.	- Selection: Not described- Performance: Assessments in the two modalities may have been done by the same team- Exclusion: Some images were not evaluated: inadequate or lost data- No use of a gold standard
Poca et al. 2004 [35]	Radiology/Head	Images: n = 160 teleradiological examinationsAssessors: n = not mentionedA neuroradiologist and the neurosurgeon on call.	**Procedure:** The first 90 images were evaluated by the neuroradiologist and neurosurgeon on call independently.Later, images were assessed mostly by the neurosurgeon.**Outcome:**- Proportion of patients who received a tomographic examination.- Proportion of referrals to a level 3 hospital.- Mode of transferal (conventional versus medicalized ambulance)**Instrument:**Not mentioned	Increase in tomographic examinations from 15% in 1997, when telemedicine was not available to 22% in 1998, when telemedicine was available.Decrease in the number of patients transferred to a level 3 hospital from 14% in 1997 to 7% in 1998. Increase in the number of patients treated at the referring hospital (27% in 1997 and 34% in 1998). Unnecessary transfers were avoided.Increase in number of patients referred with medicalized ambulances, when telemedicine was available.	- No statistical tests- No use of a gold standard
Pap et al. 2002 [32]	Clinical image and radiology/Plastic surgery	Images: n = 20From 20 patients with 12 hand injuriesAssessors: n = 4Attending plastic surgeons	**Procedure:**The assessors reviewed the digital images together with a telephone call.**Outcome:**Descriptive data on management decision.**Instrument:**Not mentioned	The initial management suggested by the resident was modified on some occasions, particularly with complex problems.	- Selection: Convenience sampling, although in random order- No statistical tests- No use of a gold standard
Ricci et al. 2002[43]	Radiology/General trauma	Images of 108 patients with 123 acute fracturesAssessors: n = not mentionedAttending orthopaedic surgeon	**Procedure:**For each injury, 3 treatment plans were formulated and recorded after: 1) traditional verbal communication. 2) digitized images were reviewed. 3) review of the original radiographs and physical examination.**Outcome:**Treatment plans formulated after each step. Two different types of deviations from the original plan were distinguished:1. Changes in the acute management - any emergency department procedures, emergent operative procedures, or dispositions that were not part of the original plan.2. Changes in the ultimate management - changes to the original plan that did not affect emergency department treatment, emergent operative procedures, or the disposition of the patient.**Instrument:**Standardized data intake form.	26/123 (21%) plans were changed after viewing the radiograph images (12 acute management and 14 ultimate), but none were changed after viewing the original radiograph.In 27/123 (22%) cases the attending physician thought that review of images would be helpful to determine an accurate treatment plan:In 15/27 (56%) cases plans were changed (7 acute management and 8 ultimate).In the 96 fractures were images were not thought to be helpful, 11/96 (11%) plans were changed (5 acute management and 6 ultimate)	- Selection: Convenience sampling- Performance: An author was the assessor- No statistical tests- No use of a gold standard
**Post-implementation (Implemented system)**					
Moya et al.2010 [44]	Radiology/Head	39 consultations(from 7 referring hospitals)Assessors: n = not mentionedNeurosurgeons	**Procedure:**Assessment before and after viewing the Web-based images.**Outcome:**Change in transfer and management decision.**Instrument:**Three questions on the Web site.**Statistics:**Binominal distribution to calculate the 95% CI.Fisher's exact test was used to compare recommended management changes between those who were transported and not.	Before viewing the images, 25/39 (64%) would have been accepted for transport.After viewing the images, 14/39 (36%) resulted in transfer.44% (11/25) of the transports were avoided and the patients were managed locally.The neurosurgeons recommended management changes in 44% (17/39) of all consultations.	- No use of a gold standard
Wallace et al. 2008 [25]	Clinical image and radiology/Plastic surgery	389 referrals where telemedicine was available (243 used telemedicine) and 607 where only telephone referral was availableAssessors: n = not mentionedReceiving clinicians	**Procedure:**Telemedicine assisted referrals compared to telephone only referrals.**Outcome:**Changes in patient management**Instrument:**Not mentioned**Statistics:**Chi squared test and CI calculation	Significant difference (p = 0.004) in the management of patients with and without the availability of the telemedicine system.Significantly fewer patients needed further assessment or review and more could be directly booked for definitive care on an operating list in the Day Surgery Unit, when telemedicine was available.Decrease in number of occasions when the referral hospital was unable to accept a referral due to a lack of capacity.No increase or decrease in patients being managed with only telephone advice, nor for patients admitted to their local hospital to await transfer to the referral hospital.	- Selection: The selection of hospitals and units is not described- No use of a gold standard
Wallace et al. 2007 [24]	Clinical image and radiology/Plastic surgery	389 referrals from the telemedicine-equipped units, (246 used telemedicine) and607 referrals by telephoneAssessors: n = not mentionedReceiving clinicians	**Procedure:**Telemedicine assisted referrals compared to telephone only referrals.**Outcome:**- Management of referrals (such as use of day surgery)- Accuracy of triage for those attending day surgery for hand trauma- Management of patients with burn injuries**Instrument:**Not mentioned (used 9 management options)**Statistics:**Chi squared test and CI calculation	Overall use of day surgery showed an 11% increase in use. 28% for telemedicine available compared to 17%.Reduction in unnecessary attendance for hand trauma surgery (13% in telephone referrals vs. 3% in telemedicine referrals).The burns unit and day surgery unit demonstrated a significantly improved accuracy of triage.	- Selection: The selection of hospitals and units is not described- No use of a gold standard
Goh et al.1997 [42]	Radiology/Head	Images of 35 patients referred with teleradiology28 patients referred by telephoneAssessors: n = not mentionedNeurosurgeons	**Procedure:**Neurosurgeons reviewed cases either by telephone consultation or by telephone consultation together with transmitted images.**Outcome:**- Transfer time from the telephone decision to arrival at the neurosurgical unit.- Comparison of therapeutic intervention – additional measures advised by the neurosurgeon prior to transfer.- Comparison of secondary insults – adverse events that could affect the outcome.**Instruments:**Not mentioned**Statistics:**Fisher's exact	Therapeutic interventions prior to the transfer occurred in 3/28 patients (10.7%) in the group without teleradiology, and in 10/31 patients (32.1%) in the teleradiology group (p = 0.062).Incidence of secondary insults (adverse events) occurred in 9/28 patients (32.1%) in the group without teleradiology, and in 2/31 patients (6.4%) in the teleradiology group (p = 0.017).	- No use of a gold standard

None of the studies used a gold standard, and nine studies did not use statistical tests [22], [31], [32], [34]–[36], [39], [40], [43]. The selection of cases was often not clearly described or explained [22], [24], [25], [29], [40], [41], and four studies employed convenience sampling [32], [34], [36], [43]. Furthermore, the performance of some studies was not clarified [22], [36], [40], [41], [43] (Table 4).

Clinical outcome was assessed only in one of the recent studies [42]. In this article, mortality and Glasgow Outcome Score (GOS) at 6 months were compared between the patients who were transferred following telephone consultation and those transferred following telemedicine consultation (including images). Proportion of poor outcome (dead, vegetative or severely disabled) was higher in the group without telemedicine (32,1% vs 25,8%), but these differences were not significant. Overall mortality in both groups was the same (14,3%).

## Discussion

### Main findings

This review dealt specifically with systems based on the transfer of images as a mean of consultation on acute injuries of various kinds. To date, by and large, those systems use above all radiologic images, they are evaluated at a feasibility stage or during small-scale pilot testing, and are put in place in a limited number of countries, all of which are high income. None of them are pre-hospital.

Whereas the impact of the systems on diagnostic validity and management outcomes are often studied (see below). As is the case in other fields of telemedicine, the data at hand are less informative regarding both system quality and user satisfaction and we found only anecdotal economic evaluations [22]–[24], [31] although methodological examples are available in the literature [46]. This may be due in part to the short life of some of the systems evaluated, but these knowledge gaps are now regarded as research areas to receive priority so as to allow policymakers and health care planners to make informed decisions (not least in low- and middle-income countries) [47]–[49]. Partly as a consequence, no standard methods of measurement emerge as regards systems quality or user satisfaction. From the reports available on user satisfaction for instance [24], [25], [31], [32], [36], ease of use and usefulness of telemedicine are the two aspects studied and the studies are difficult to reproduce.

As observed in previous reviews concerned with telemedicine for the support of medical care in general [1]–[9] or in some reviews for emergency care of injuries in particular [15], [16], the quality of the evaluations performed to date is somewhat poor, sometimes by using inadequate or no gold standard or an imprecise reference to validate the system, sometimes by their limited sample size and inappropriate statistical methods, and sometimes even by their poor reproducibility.

### Diagnostic validity

Whether image-based telemedicine in acute care yield accurate clinical diagnosis was investigated for 16 systems and the majority relied solely on radiological images, some for injuries in general and others for specific body parts (e.g., hand or head). All but two feasibility studies [29], [38] involved accuracy assessments. They were of varying size in terms of number of cases and a gold standard was used in all instances. Evaluations of systems at the small-scale phase [22], [23], [31], [32], [35], [39]–[41] were more inclined to use a gold standard over time and in particular when they were of larger size (number of cases/images). The implemented system evaluated [42] did not use a gold standard.

Not surprisingly, the general impression is that transmitted images, above all radiological ones and of a variety of body parts, can be accurately interpreted by specialists and that this has become more evident over time, i.e. while the technology itself allowed for better pictures, transmission and reading conditions. This finding can be interpreted as if consulting a radiologist or specialist is (has become) as accurate when using transmitted images as when using original ones. It is also of note that factors like age and experience of the teleexpert may impact on the level of accuracy just as do some characteristics of the injury.

### Management outcomes

Whether telemedicine affected patient management was investigated for 15 systems and all but one of them implied radiological images, some for injuries in general and others for specific body parts (e.g., hand or head). At the feasibility stage, three systems [29], [36], [45] out of 10 were assessed for their potential influence in that respect. All small scale system implementations assessed patient management (9 systems; [22], [31], [32], [34], [35], [39]–[41], [43]) and so did the three system evaluated once roll out (4 articles) [24], [25], [42], [44].

The general impression is that consultation by telephone contributes to a change in management plan, including accuracy of triage/referral or a given treatment plan/procedure. This can be interpreted as if consulting a radiologist or specialist influences the management decisions made in acute care regarding injured patients. This in turn is conditional to transmitted images being as accurately interpreted as original ones (in case of radiology) or as at seeing the patient at bedside. Some of the data supporting this finding are perceptual (point of care or expert) and others are factual – a change was reported/observed.

The data at hand however is of relatively poor quality with limitations in the use of gold standard, in study size (8 evaluations being based on less than 50 cases 2 having between 80 and about 100 and the remaining 4 having over 150 cases), and in statistical methods.

The review was limited in the way that most studies came from high-income countries and may not be representative of the conditions prevailing in low- and middle-income countries where this kind of research is much needed [47], [50]. The research is based on well-established databases commonly used in similar type of reviews. We may have missed some studies captured in other types of databases but we assume that this loss is most likely to be small given the broad scope of the search itself. Furthermore, the review is restricted to articles written in the English, French, Spanish, German or Nordic languages. We also acknowledge the high likelihood of publication bias in favour of studies showing positive effect of telemedicine systems, which affects the state of knowledge [47]. Unfortunately, we were not able to describe and compare the technical features of the systems as much as we had expected in the beginning of the review process. The studies were published in different type of journals, but mainly medical ones, and the level of detail was very uneven. It goes without saying that it would be a great contribution to this field of research – and practice – if there were clear criteria to be met for the description of the systems evaluated.

### Way forward

As availability of telecommunication and information technology expands, and penetration into low- and middle- income countries increases, image based telemedicine can play a key role in increasing access to expert advice in the acute care of injured patients. However, current evidence is generally of low methodological quality and is limited in focus. In order to facilitate scale up of injury based acute care telemedicine systems – in a time of increasing burden of injury in many parts of the world – the literature is still incomplete.

- Studies are needed to inform program development and implementation in general (to better understand barriers to large-scale implementation) and in resource poor settings in particular (where such systems are most urgently needed) [48], [50], [51].

- Research in this field needs to pay greater attention to user perspective (both healthcare professionals and patients) [48], [51]. Failure to do so is a major threat to sustainability, as user acceptance is a prerequisite to implementation.

- Other aspects of telemedicine must be studied. Lack of basic evidence such as cost-effectiveness [47], [50]–[53], effect on quality of care [48], [52] and health outcome [47], [50]–[53] has been highlighted as one of the major barriers to scaling up such programs.

For scaling up telemedicine programs, several authors emphasize the importance of a common architectural design and interoperability of initiatives into existing health services [47], [50]–[53]. National policies to ensure patient security and liability [51], [53] and liaison of public and private partnerships [50]–[54] are other important elements for a broadening of initiatives. Policies could also ensure that strategies for monitoring and evaluation are included in the planning [52]. The creation of standard methods, instruments and measures would greatly assist interoperability and reproducibility of the myriad programs in use and being developed.

## Conclusions

The present systematic review shows that image-based telemedicine systems for injury emergency care tend to support valid diagnosis and influence patient management. However, the current evidence is generally of low methodological quality and is limited in focus. User and system quality aspects are poorly documented, both of which affect scale up of such programs. Further work is required on quality, interoperability, and scalability.

## Supporting Information

Checklist S1 PRISMA Checklist.
(DOC)
